# An Atypical Presentation of Myopericytoma in Palmar Arch and Review of the Literature

**DOI:** 10.1155/2014/759329

**Published:** 2014-10-02

**Authors:** Adnan Kara, Mert Keskinbora, Mahmut Enes Kayaalp, Ali Şeker, Mehmet Erdil, Murat Bülbül

**Affiliations:** Department of Orthopaedics and Traumatology, Faculty of Medicine, Istanbul Medipol University, Istanbul, Turkey

## Abstract

*Introduction*. Myopericytoma is a very rare perivascular tumor that can be presented with painful mass in lower extremities. We aimed to present an atypical presentation and location of myopericytoma. *Presentation of Case*. An 18-year-old otherwise healthy individual was admitted to outpatient clinic with complaints of numbness and pain in his right hand. He has had no trauma. On volar aspect of his right hand, a well-circumscribed, painful mass was palpated. MRI results were related to hemangioma. Surgical excision was planned and performed. Pathological investigation revealed the mass is myopericytoma. *Discussion*. This case demonstrates a rare location and presentation of myopericytoma. Reviewing the literature, discussion was made to expand the horizon for diagnosis and treatment of patients with similar symptoms. *Conclusion*. Myopericytoma can rarely present with numbness and pain in affected region. Surgical excision is helpful for definitive diagnosis and symptom relief.

## 1. Introduction

Myopericytomas are perivascular benign neoplasms. These uncommon lesions are believed to arise from perivascular myoid cells [1]. They are seen most frequently in lower extremities in the dermal or subcutaneous location [1–3]. A myopericytoma patient typically presents with well-circumscribed, slow growing, painless firm mass [2].

In this paper, we present 18-year-old male patient with a painful mass in thenar region of his right hand. He had also numbness in ulnar side of his thumb. We aimed to discuss this atypical presentation of this rare entity in the light of the literature.

## 2. Case Report

**Figure 1:**
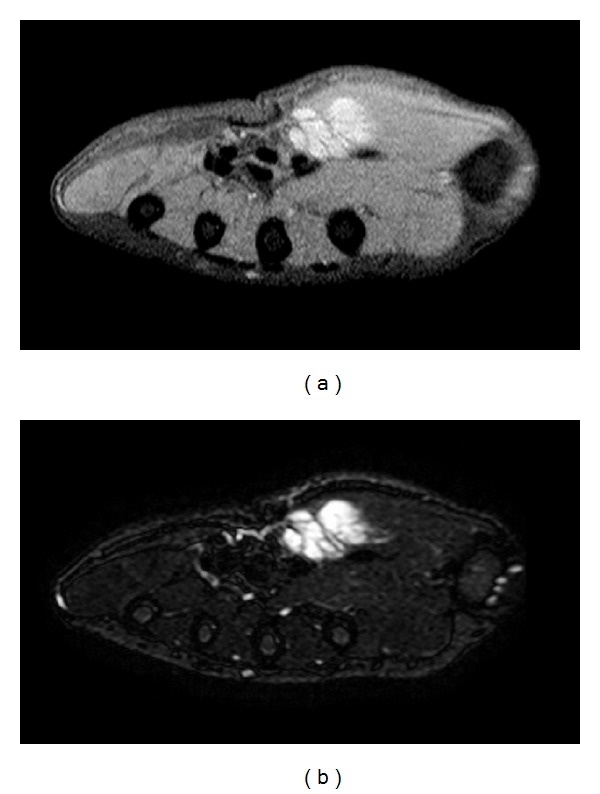
(a) Low signal intensity in T1A. (b) High signal intensity in T2A.

An 18-year-old male patient was admitted to our outpatient clinic with complaints of ongoing pain in his thenar region and numbness of 6-month duration in ulnar side of his right thumb. In the physical examination, a 3 × 2 cm well-circumscribed, painful mass in his thenar region was seen. His past medical history and family history were unremarkable. Preoperative complete blood count, erythrocyte sedimentation rate, and C-reactive protein values were within normal ranges. Preoperative X-ray examinations were normal. Preoperative MRI demonstrated a lobulated, well-circumscribed, contrast enhanced mass, which was 24 × 20 × 16 mm in size and had low signal intensity in T1A (Figure 1(a)) and high signal intensity in T2A (Figure 1(b)). These findings were found relevant to hemangioma.

**Figure 2:**
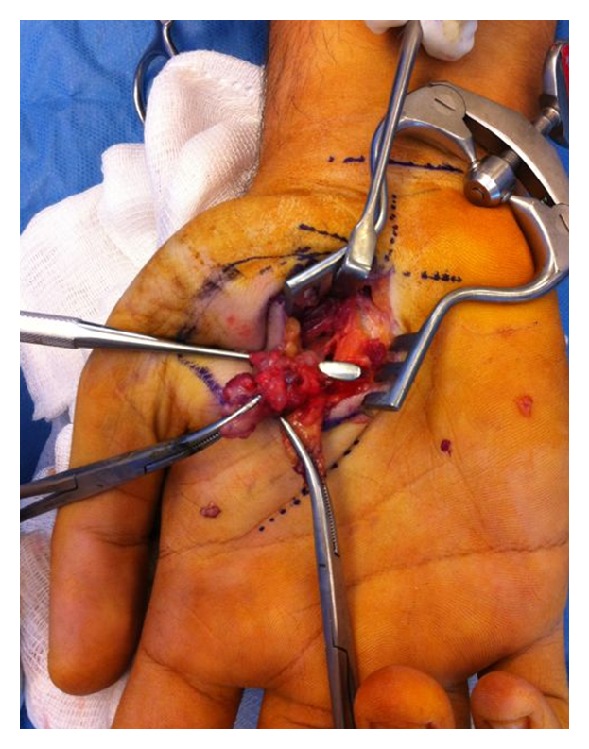
Lesion was excised with microscopic dissection.

Under tourniquet control, we perform a curved incision over the mass in the thenar region of the right hand. With meticulous dissection of soft tissues, the margins of the lesion were exposed between adductor pollicis and flexor pollicis brevis muscles. Superficial palmar arch was also exposed during the dissection. Under the lesion, we observed the compression of the lesion to the ulnar side of palmar digital nerve with some adhesions to the lesion. We excised the lesion from the nerve under microscopic dissection (Figure 2).

The excised lesion was prepared for histopathological evaluation.

Patient reported relief of pain and numbness symptoms at the 4th week of the surgery. At the last follow-up of the 14th month after surgery, the patient was well with no recurrence or symptoms.

## 3. Discussion

Myopericytomas are uncommon benign perivascular neoplasms that show a hemangiopericytoma-like vascular pattern [1, 2, 4, 5]. Patients with myopericytoma are frequently presented with well-circumscribed, slow growing, painless firm mass [2, 3]. In contrast, our patient complained of a painful lesion in his right hand with numbness radiating to the ulnar side of his thumb. We observed the compression of the lesion to the ulnar side of palmar digital nerve during dissection. At the 4th week of surgery, numbness and pain complaint were relieved.

These lesions frequently arise in lower extremities, whereas they can be found in upper extremities, head and neck region, and the trunk [1, 4]. To the best of our knowledge, there are only two cases of hand localization of myopericytoma [6, 7]. In our patient, the lesion was in the thenar region of his right hand.

According to the literature, preoperative MR and ultrasound investigations are found to be insufficient or misleading [6, 8]. Excisional biopsy and histological examination are necessary for definitive diagnosis. Also in our case, preoperative MRI reported the case as hemangioma.

**Figure 3:**
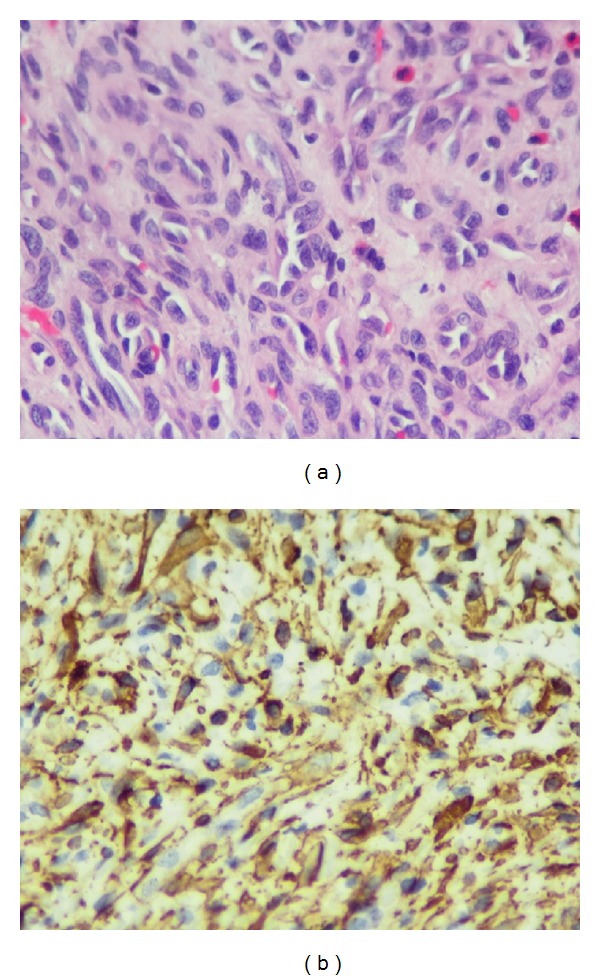
(a) Thin-walled vessels with a concentric, perivascular gathering of spindled myoid tumor cells (HE-Stain, ×40). (b) Pericytes stained positive for caldesmon.

The histopathological examination revealed CD34 expression in endothelial cells and thin-walled vessels with a concentric, perivascular gathering of spindled myoid tumor cells as similarly described in the literature (Figure 3(a)). These cells stain positive for SMA (smooth muscle actin) and often also for caldesmon, are immune negative for desmin, and are accepted as modified smooth muscle cells [1, 6] (Figure 3(b)). The same results were obtained in our case. Ultrastructurally, myopericytes have a transitional phenotype and are intermediate cells between pericytes and vascular smooth muscle cells [9].

Recurrent and/or malignant cases of myopericytomas are rarely reported, even though they are excised marginally or incompletely. Additionally, some local recurrences are reported in a few numbers [4, 5, 10–12]. In our case, no proof of recurrence was noticed at the 14th month of follow-up.

Surgical excision is the preferred treatment method. Several studies show good-to-excellent results with low rate of local recurrence [4, 7]. In our case, we performed surgical excision with meticulous dissection of nerve and vessels.

## 4. Conclusion

Myopericytomas are rare tumors that can show atypical presentations. Surgical excision is the gold standard for diagnosis and treatment.
